# Amyotrophic lateral sclerosis patient iPSC-derived astrocytes impair autophagy via non-cell autonomous mechanisms

**DOI:** 10.1186/s13041-017-0300-4

**Published:** 2017-06-13

**Authors:** Martin Madill, Katya McDonagh, Jun Ma, Alice Vajda, Paul McLoughlin, Timothy O’Brien, Orla Hardiman, Sanbing Shen

**Affiliations:** 1 Regenerative Medicine Institute (REMEDI), School of Medicine, National University of Ireland Galway, Galway, Ireland; 2 Anatomy Department, Hebei Medical University, Shijiazhuang, Hebei Province People’s Republic of China; 3 Academic Unit of Neurology, Trinity Biomedical Sciences Institute, Trinity College Dublin, 152-160 Pearse Street, Dublin 2, Ireland

## Abstract

Amyotrophic lateral sclerosis, a devastating neurodegenerative disease, is characterized by the progressive loss of motor neurons and the accumulation of misfolded protein aggregates. The latter suggests impaired proteostasis may be a key factor in disease pathogenesis, though the underlying mechanisms leading to the accumulation of aggregates is unclear. Further, recent studies have indicated that motor neuron cell death may be mediated by astrocytes. Herein we demonstrate that ALS patient iPSC-derived astrocytes modulate the autophagy pathway in a non-cell autonomous manner. We demonstrate cells treated with patient derived astrocyte conditioned medium demonstrate decreased expression of LC3-II, a key adapter protein required for the selective degradation of p62 and ubiquitinated proteins targeted for degradation. We observed an increased accumulation of p62 in cells treated with patient conditioned medium, with a concomitant increase in the expression of SOD1, a protein associated with the development of ALS. Activation of autophagic mechanisms with Rapamycin reduces the accumulation of p62 puncta in cells treated with patient conditioned medium. These data suggest that patient astrocytes may modulate motor neuron cell death by impairing autophagic mechanisms, and the autophagy pathway may be a useful target in the development of novel therapeutics.

## Introduction

Amyotrophic Lateral Sclerosis (ALS), a fatal neurodegenerative disease, is characterized by the progressive loss of motor neurons. Death typically occurs within 2–5 years of onset, usually due to respiratory failure [1] and currently there is no efficacious therapy available [2]. ALS is associated with mutations in a wide number of genes, with *C9ORF72* and *SOD1* mutations being the most common [3]. However, the mechanisms by which these mutations cause ALS remain elusive.

ALS is a heterogeneous condition, and defects in several molecular pathways have been identified, including oxidative stress [4], impaired axonal transport [5], glutamate excitotoxicity [6] and the secretion of toxic factors by non-neuronal cells [7]. In addition to neuronal degeneration, accumulation of misfolded ubiquitinated protein aggregates is a hallmark of most forms of ALS [8]. The compositions of these aggregates varies between ALS cases, but often comprise proteins known to be causative of ALS, including TDP-43, SOD1, p62, FUS and OPTN1, as well as dipeptide repeats generated due to a repeat expansion in the *C9ORF72* gene [3]. This suggests that impaired proteostasis may be central to the pathogenesis of ALS.

Autophagy is a cellular mechanism required for the degradation of long-lived and misfolded proteins. Autophagy is negatively regulated by mTOR, which phosphorylates p70S6K, in turn dephosphorylating ULK1, a component of the autophagy initiation complex [9]. Under certain conditions, including nutrient starvation or pharmacological treatment, mTOR may be inhibited, resulting in dephosphorylation of p70S6K, activation of ULK1 and initiation of autophagy. The autophagy initiation complex further comprises ATG13, ATG101 and FIP200 which together act to phosphorylate BECLIN-1 and activate VPS34 to induce autophagosome formation [10]. A range of other autophagy related proteins (ATG) including ATG5, ATG12, ATG7, ATG10 and AGT16-L work together to promote elongation of the developing autophagosome [11]. LC3B is a key protein involved in selective autophagy. Following conjugation of phosphatidylethanolamine to generate LC3-II, it is targeted to developing autophagosomes by interaction with ATG5-ATG12 where it subsequently recruits cargo destined for autophagic degradation into the autophagosome. An autophagy adapter protein, p62, contains a ubiquitin-binding domain in addition to an LC3B interacting domain, allowing the selective degradation of ubiquitinated proteins [12]. Completed autophagosomes are transported along microtubules by Dynein proteins to the lysosomal rich perinuclear region where SNARE proteins regulate the fusion of autophagosome and lysosome [13, 14]. Contents within the completed autophagosome, including p62, are degraded via lysosomal hydrolases and subsequently recycled back into the cytoplasm.

Several studies have implicated altered autophagy in various ALS models. Decreased ULK1 mRNA in ALS patient samples suggests an impaired initiation of autophagy [15], whereas increased LC3B and p62 accumulation indicates impaired autophagic flux [16–18]. However, the strongest evidence for a role of autophagy in the pathogenesis of ALS may arise from the function of proteins encoded by many of the genes associated with the development of ALS – the majority of them may in some way function as part of the autophagy pathway. SOD1 may activate autophagy through activation of BECLIN-1 [19]. C9ORF72 has been shown to regulate endosomal trafficking, as well as possibly interacting with LC3B [20]. Additionally, mutations in several autophagy receptor proteins, including p62, Optineurin and Ubiquilin 2, have been identified in ALS patients [3]. Furthermore, activation of the autophagy pathway by pharmacological means has been demonstrated to increase survival in ALS animal models [21–24].

In recent years, an increasing focus has been placed on non-neuronal cells in the pathogenesis of ALS. Several studies have indicated that patient astrocytes may secrete factors which are directly toxic to motor neurons [7, 25, 26]. However, the identities of these factors remain largely unknown.

We hypothesize that impaired autophagy may be central to the pathogenesis of ALS. Herein we established induced pluripotent stem cells (iPSCs) from ALS patients and age-matched healthy controls. We demonstrate that patient astrocyte conditioned medium (ACM) decreases the viability of motor neurons derived from both control and patient iPSCs. To investigate the mechanisms by which astrocytes may mediate cell death we cultured HEK293T cells with ACM from both control and patient iPSC-derived astrocytes. We demonstrate that cells treated with patient ACM show lower expression of LC3-II, a protein required for selective degradation of p62. We further show a concomitant accumulation of p62 puncta, suggesting impaired autophagic flux. Additionally, we demonstrate increased accumulation of SOD1, the most widely studied protein in relation to ALS pathogenesis. These results indicate that patient astrocytes mediate an imbalance in the autophagy pathway, which may result in the accumulation of ALS-related proteins, causing pathological effects.

## Results

### Generation and characterization of ALS patient and control iPSCs

To investigate cellular pathology and disease mechanisms of ALS, we recruited skin punches from healthy volunteers and ALS patients (Table 1). Two ALS patients carry the G_4_C_2_ hexanucleotide repeat expansion of the *C9ORF72* gene whereas the third patient is negative for the expansion. Although one healthy control is younger, two other controls match the age of two patients precisely.

**Table 1:** Details of patient and control iPSC lines used in this study

iPSC line	Age	Sex	Patient status	Reprogramming method
iPS04c3	20	Male	Control	Lentiviral
iPSC1cx1	57	Female	Control	Episomal
iPSC3c2	47	Female	Control	Episomal
iPS21c1	45	Male	ALS C9ORF72 R.E.	Lentiviral
iPS21cx	45	Male	ALS C9ORF72 R.E.	Lentiviral
iPS24c1	61	Male	ALS, C9ORF72 negative	Episomal
iPS31c8	57	Female	ALS C9ORF72 R.E.	Lentiviral

Two patients harbor a G_4_C_2_ hexanucleotide repeat expansion in the *C9ORF72* gene

**Fig. 1:**
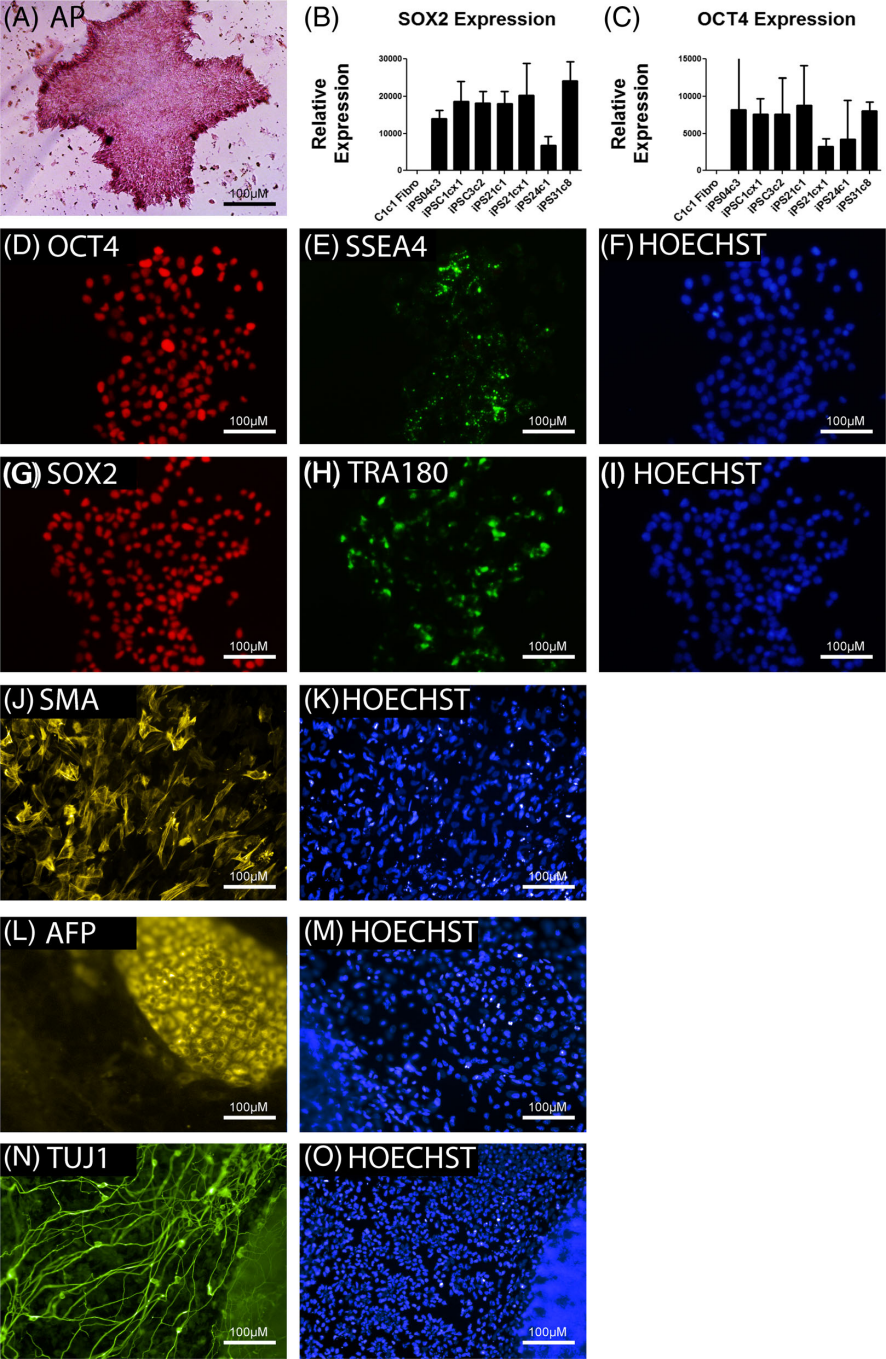
Validation of pluripotency in reprogrammed cells. **a** Representative image of alkaline phosphatase (AP) staining of iPSCs. **b**, **c** RT-PCR analysis of *OCT4* and *SOX2* expression in patient and control iPSC lines. Expression was normalized to GAPDH expression. **d**, **e**, **f** Immunocytochemistry analysis of OCT4 and SSEA4 in iPSCs and the corresponding Hoechst stain. **g**, **h**, **i** Immunocytochemistry analysis of SOX2 and TRA180 in iPSCs and the corresponding Hoechst stain. **j**, **l & n** Expression of Smooth Muscle Actin (SMA), Alpha Fetal Protein (AFP) And Beta Tubulin (TUJ1) confirmed tri-lineage differentiation potential of iPSCs with respective Hoechst stains shown in (**k**), (**m**) and (**o**). Scale bars 100 μm

Cells were first characterized as pluripotent by demonstrating strong staining of Alkaline Phosphatase (AP), with particularly high expression observed at the perimeter of the expanding iPSC colonies (Fig. 1a). Following this, RT-PCR analyses were performed to demonstrate greatly increased expression of endogenous *OCT4* and *SOX2*, canonical pluripotent stem cell markers (Fig. 1b, c). Dermal fibroblasts were used as a negative control for expression of these markers. To compliment the RT-PCR analyses, immunostaining was performed to demonstrate expression of SOX2 and OCT4, as well as stem cell surface markers SSEA4 and TRA180 in iPSCs (Fig. 1d, e, g and h). Nuclei were stained with Hoechst to indicate the majority of cells stained positive. Finally, iPSCs were differentiated through an embryoid body stage and spontaneously differentiated to cells of all three germ layers. Cells positive for Smooth Muscle Actin (SMA), Alpha Fetal Protein (AFP) and β-Tubulin (TUJ1), markers of mesodermal, endodermal and ectodermal lineages respectively, were all identified, indicating tri-lineage differentiation potential of iPSCs (Fig. 1j, l and n). Again, nuclei were stained with DAPI to identify total cells. These data confirm the pluripotency of our control and patient derived iPSCs.

### Patient iPSC-derived astrocyte conditioned medium is toxic to iPSC-derived motor neurons

**Fig. 2:**
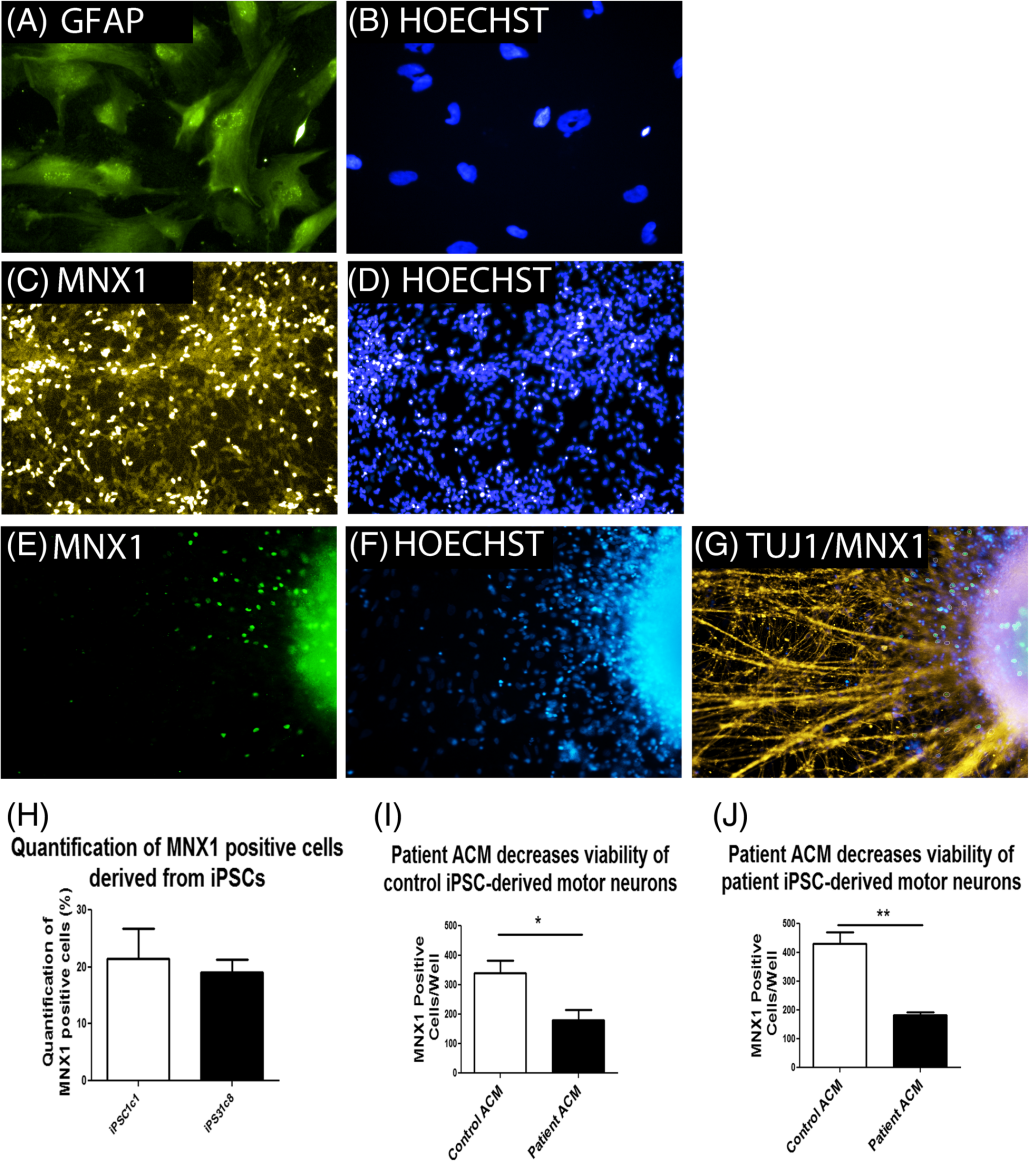
Patient ACM decreases viability of control and patient iPSC-derived motor neurons. Astrocytes and motor neurons were generated from iPSCs. **a** Representative image of GFAP staining confirms the generation of astrocytes from MNPs, with corresponding DAPI stain shown in (**b**). **c** Representative image of anti-MNX1 staining confirmed the generation of motor neurons derived from iPSCs with corresponding DAPI stain shown in (**d**). For quantification of motor neuron survival motor neurons were cultured in the presence of ACM for 5 days, after which cells were fixed and stained for anti-MNX1 (**e**), DAPI (**f**) and TUJ1 (**g**). **h** Quantification of motor neurons derived from control and patient iPSC lines iPSC1c1 and iPS31c8. **i** Quantification of control iPSC1c1-derived MNX1 positive motor neurons after 5 days of culture with control (*n* = 3) and patient (*n* = 4) ACM. **j** Quantification of patient iPS31c8-derived MNX1 positive motor neurons after 5 days of culture with control (*n* = 3) and patient (*n* = 4) ACM. *p < 0.05 and **p < 0.01 were considered statistically significant. *Error bars* represent standard error of mean. *Scale bars* 100 μm

ALS is characterized by the progressive loss of motor neurons, with glial cells recently being implicated in disease pathogenesis. It has repeatedly been demonstrated that glial cells may secrete factors which are directly toxic to motor neurons [7, 26–29]. As such, we aimed to examine whether the same phenotype could be recapitulated with our patient derived cells. Firstly, iPSCs were differentiated to motor neuron progenitor cells (MNPs), and subsequently motor neurons, using a recently described protocol [30]. The motor neuron identity was confirmed by staining with anti-MNX1, a marker of motor neurons (Fig. 2c). Additionally, to generate glial cells, MNPs were cultured in medium containing 10% FBS. This resulted in the generation of cell populations with large fibroblastic morphology which stained positive for GFAP, a marker of astrocytes (Fig. 2a). After passaging cells, the morphology was uniformly fibroblastic and 100% of cells examined stained positive for GFAP.

To investigate a toxic effect of astrocytes on motor neurons, we cultured motor neurons derived from one patient (iPS31c8) and one control (iPSC1c1) with astrocyte conditioned medium (ACM) from three patient and three control iPSC-derived GFAP^+^ astrocyte populations. After 5 days of culture MNX1 positive cells were quantified via automated image analysis. We observed a significant decrease in motor neuron viability in the presence of ACM from patient derived cells relative to ACM from control cells (Fig. 2i, j). This suggests that astrocytes may secrete factors which directly affect the generation or survival of iPSC-derived motor neurons in vitro.

Interestingly, this phenotype has been shown to be specific for motor neurons, with other cell types, including GABAergic neurons and dorsal root ganglion neurons, demonstrating no decrease in viability [31]. However, despite several studies showing similar decreases in motor neuron viability, few have identified possible factors which may contribute to this decrease.

### Patient ACM impairs autophagic flux in HEK293T cells

Impaired autophagy has been widely implicated in the pathogenesis of ALS [32]. Mutations in several autophagy-related genes may result in the development of ALS [33]. Indeed, C9ORF72, mutations of which are the most common cause of ALS, regulates the expression and activity of ULK1, thereby regulating initiation of autophagy [34]. Furthermore, activation of autophagic pathways has been shown to alleviate the pathogenesis of ALS [21, 22, 24]. Despite these findings, the precise mechanisms by which autophagy is impaired and may affect motor neurons are unknown.

**Fig. 3:**
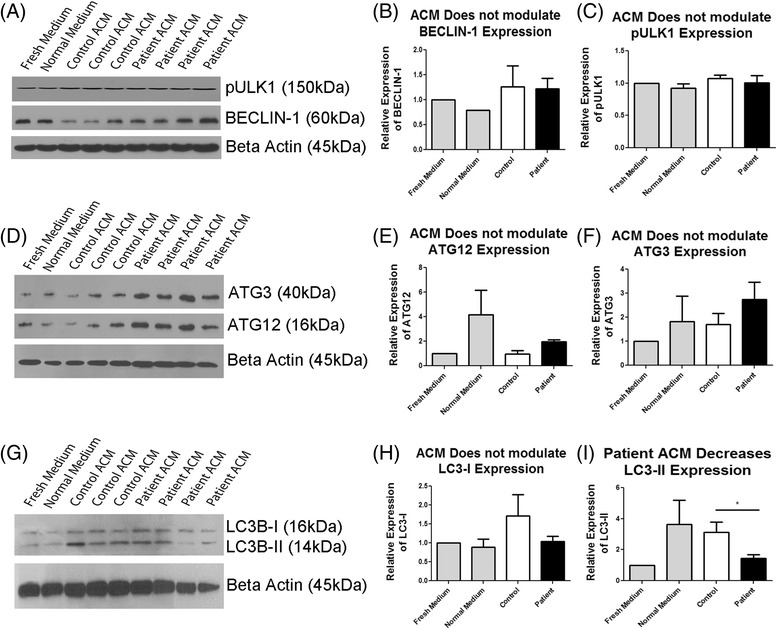
ACM alters expression of autophagy related proteins in HEK293T cells. HEK293T cells were cultured in ACM from control (*n* = 3) and patient (*n* = 4) iPSC-derived astrocytes for 5, after which protein was harvested for western blot analysis. Cells were also cultured in DMEM + 10% FBS with daily media changes (Fresh medium) and DMEM + 10% FBS without changing the medium for 5 days (Normal Medium). **a**, **d**, **g** Western blot analyses of pULK1, BECLIN-1, ATG3, ATG12, LC3-I and LC3-II. **b**, **c**, **e**, **f**, **h**, **i** Densitometry of western blot analyses. All values were normalized to β-Actin expression. *p < 0.05was considered statistically significant. *Error bars* represent standard error of mean

Interestingly, it has been demonstrated that glial cell conditioned medium may have pro-autophagic effects in an animal model of Huntington’s disease [35]. We hypothesized that ACM from ALS patient and control iPSC-derived astrocytes may differentially regulate autophagy. To investigate ACM mediated effects on the autophagy pathway we cultured HEK293T cells with ACM from patient and control astrocytes. As autophagy may be induced by nutrient starvation, we additionally cultured cells with normal medium for 5 days without replenishing, and cells for which medium was replenished daily, for comparative purposes. No difference in cell viability was observed between cells treated with control and patient ACM, as determined by LDH and MTS assays. After 5 days, protein lysates were harvested and western blot analyses were performed to investigate expression of autophagy-related proteins (Fig. 3). No changes in BECLIN-1, pULK1, ATG3 or ATG12 were observed (Fig. 3a-f), suggesting no alterations to autophagic initiation in cells treated with ACM. We further examined levels of LC3-I, and its lipidated and activated form, LC3-II, in response to ACM. Cells treated with patient ACM were shown to express significantly lower levels of LC3-II, suggesting decreased autophagic flux (Fig. 3g-i).

**Fig. 4:**
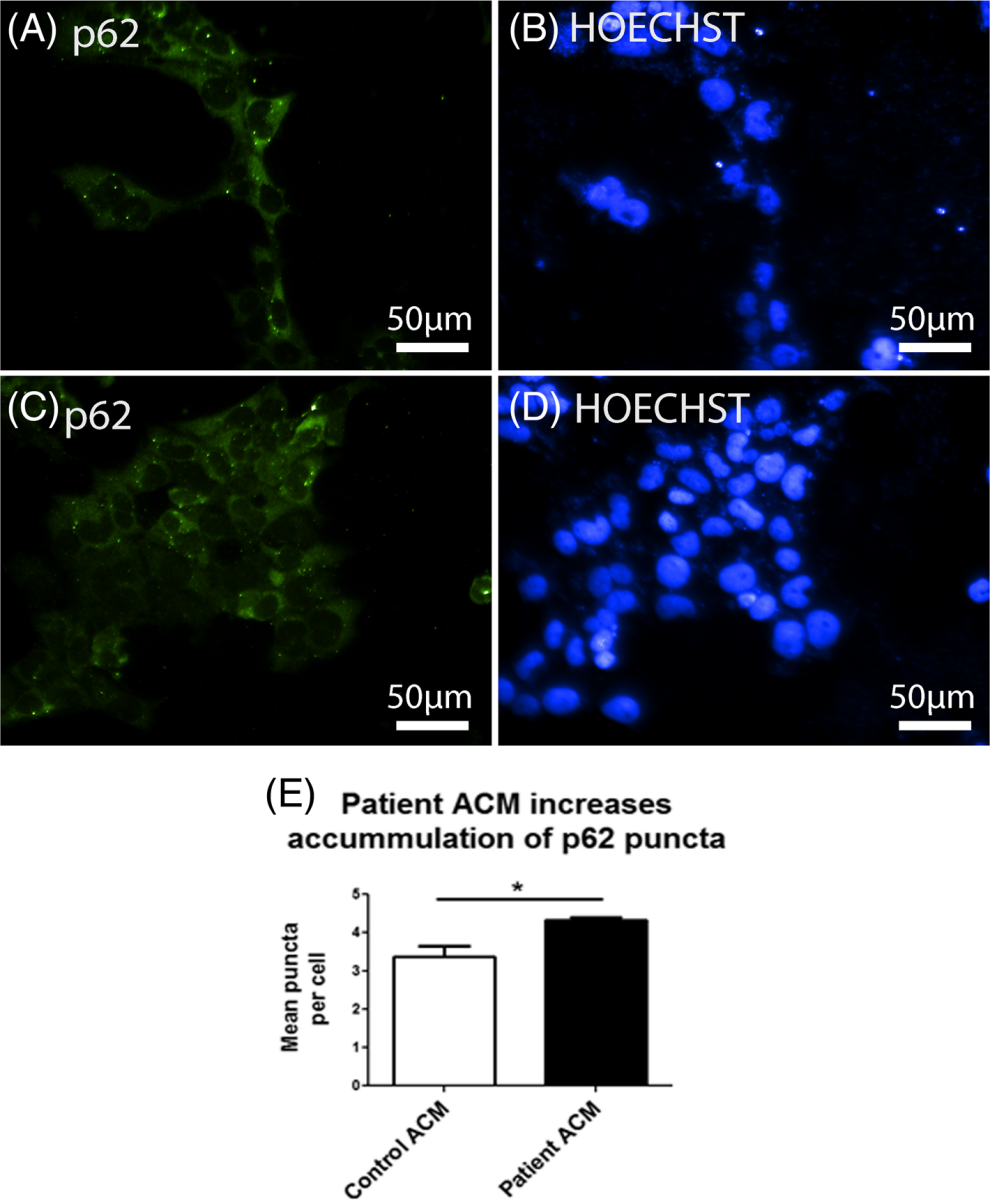
Patient ACM increases accumulation of p62 puncta in HEK293T cells. HEK293T cells were treated with ACM from control and patient iPSC-derived astrocytes for 5 days and subsequently fixed in 4% paraformaldehyde and stained for p62 expression. p62 puncta were quantified using automated image analysis. **a** Representative image of p62 expression in HEK293T cells treated with control ACM (*n* = 3), with Hoechst staining shown in (**b**). **c** Representative image of p62 expression in HEK293T cells treated with patient ACM (*n* = 4), with Hoechst staining shown in (**d**). **e** Quantification of p62 puncta in cells treated with ACM. *p < 0.05 was considered statistically significant. *Error bars* represent standard error of mean

**Fig. 5:**
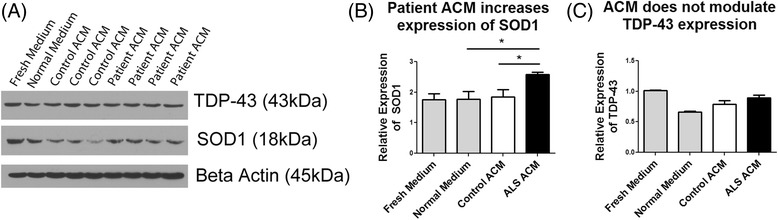
Patient ACM increases accumulation of SOD1. HEK293T cells were cultured in ACM from control (*n* = 3) and patient (*n* = 4) iPSC-derived astrocytes for 5 days, after which protein was harvested for western blot analysis. Cells were also cultured in DMEM + 10% FBS with daily media changes (Fresh medium) and DMEM + 10% FBS without changing the medium for 5 days (Normal Medium). **a** Western blot analysis of SOD1 and TDP-43. **b**, **c** Densitometry analyses of SOD1 and TDP-43 expression. All values were normalized to β-Actin expression. *p < 0.05 was considered statistically significant. *Error bars* represent standard error of mean

To further investigate autophagic flux in these cells immunocytochemistry was performed to analyze p62 accumulation (Fig. 4). After 5 days of culture in ACM cells were fixed and stained for p62 expression and the number of puncta quantified using automated image analysis. We observed a significant increase in p62 puncta in cells treated with patient ACM relative to cells treated with control ACM (Fig. 5e). This data, in combination with western blot results showing decreased LC3-II expression, indicates decreased autophagic flux in response to patient ACM.

A recent study has demonstrated increased accumulation of p62 in iPSC-derived motor neurons from patients harboring the G_4_C_2_ C9ORF72 repeat expansion [36]. These cells similarly demonstrated no change in LC3-II levels, whereas iPSC-derived cortical neurons from the same patients showed increased LC3-II. We suggest that patient astrocytes mediate this impaired autophagic flux in target cells, and may contribute to the pathogenesis associated with ALS.

### Patient ACM increases SOD1 expression

p62, and thus autophagic flux, is required for the degradation of several ALS related proteins, including SOD1 and TDP-43 [37, 38]. To further investigate the pathologic mechanisms associated with ALS patient ACM, we investigated the expression of ALS-related proteins SOD1 and TDP-43, two of the most common proteins associated with the pathogenesis of ALS (Fig. 5). Significantly higher SOD1 expression was identified in cells exposed to patient ACM (Fig. 5b). This increase in SOD1 may be due to impaired autophagy, decreasing the turnover of SOD1. In patients who harbor SOD1 mutations, this increase in expression would likely be detrimental to motor neuron survival. However, it remains to be confirmed whether astrocytes harboring SOD1 mutations would similarly disrupt autophagic flux.

Conversely, no change in TDP-43 was observed (Fig. 5c). However, it should be noted that our experiments were relatively short in duration, only covering a 5-day period. It has been demonstrated that TDP-43 may be cleared by activation of the autophagy pathway [39]. As the majority of ALS patients present with TDP-43 pathologic inclusions it would be interesting to observe whether prolonged impairment of autophagy via patient ACM results in progressive accumulation of this protein. More studies will be warranted to investigate the effects of ALS patient ACM on ALS-related proteins.

### Activation of autophagy prevents increased accumulation of p62

**Fig. 6:**
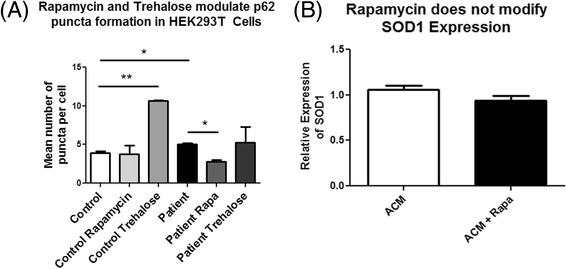
Rapamycin alleviates p62 accumulation in cells treated with patient iPSC-derived ACM. HEK293T cells were treated with control (*n* = 3) and patient (*n* = 3) ACM for 5 days, with either 200nM Rapamycin or 100nM Trehalose for the final 2 days where indicated. Cells were then fixed in 4% paraformaldehyde and stained for p62 (**a**), or protein harvested to assess changes in SOD1 expression (**b**). P62 puncta were analyzed using automated image analysis. Trehalose increased p62 puncta in HEK293T cells treated with control ACM, but not patient ACM. Rapamycin decreased levels of p62 puncta in HEK293T cells treated with patient ACM, but had no effect on cells treated with control ACM. Rapamycin had no effect on SOD1 levels following treatment with ACM. *p < 0.05 and **p < 0.01 were considered statistically significant. *Error bars* represent standard error of mean

Activation of autophagic mechanisms has been shown to be beneficial in cellular and animal models of ALS [22, 24, 39]. As such we aimed to investigate whether Rapamycin or Trehalose, known activators of autophagy, could prevent the increased accumulation of p62 puncta in response to patient ACM (Fig. 6a). We treated cells with ACM in addition to either Rapamycin or Trehalose. Trehalose was shown to have an opposite effect as anticipated, which greatly increased the accumulation of p62 puncta in control ACM-treated cells, but had a high variability in patient ACM-treated cells. Rapamycin had no effect on the accumulation of p62 puncta in cells treated with control ACM, However, decreased the accumulation of patient ACM-induced p62 puncta, returning to levels similar to cells treated with control ACM. We suggest that Rapamycin may be a useful compound to counter the effect of impaired autophagic flux inferred by astrocytes in ALS patients. Further, more comprehensive analysis of the mechanisms by which patient ACM deregulates autophagy may yield insight into novel therapeutic targets for improved treatment of ALS.

We additionally investigated whether Rapamycin could decrease the accumulation of SOD1 in cells treated with patient ACM (Fig. 6b). We found that Rapamycin had no effect on SOD1 expression. It is possible that the limited treatment with Rapamycin is insufficient to modulate SOD1 expression following treatment with patient ACM.

## Discussion

The majority of ALS research using animal models during the previous two decades has focused on *SOD1* transgenic mouse models. However, SOD1 mutations account for only ~2% of ALS cases [40]. Notably, there is accumulating evidence suggesting that *SOD1* mutations may give rise to ALS cases with divergent pathology relative to other mutations. Whereas the vast majority of ALS cases present with TDP-43 inclusions, patients harboring *SOD1* mutations are characteristically devoid of these inclusions [41]. iPSC-derived astrocytes and motor neurons offer the opportunity to investigate glial-neuronal interactions, and impairments therein, which may be attributed to a wide variety of genetic mutations and will be highly advantageous in elucidating the precise molecular mechanisms leading to the development of ALS.

This research demonstrates ALS patient ACM decreases the viability of motor neurons, consistent with previous reports [25]. This has been recapitulated using both animal model derived cells and patient derived cells harboring *SOD1* and *C9ORF72* mutations, as well as cells from patients with unknown genetic defects [7, 27]. This intriguing pathogenic mechanism offers the potential to identify novel therapeutic targets. However, the precise factors which induce motor neuron toxicity remain to be elucidated. Increased expression of prostaglandin D2 was identified as a possible culprit, but inhibition of this protein offered only mild improvements in motor neuron survival [42]. More recent evidence has suggested a complex comprising α2-Na/K ATPase/α-adducin as responsible for motor neuron cell death in a mtSOD1 mouse model [29]. Knockdown of this complex in astrocytes, or its pharmacological inhibition, is sufficient to rescue motor neurons from astrocyte mediated cell death. However, this has only been demonstrated in *SOD1* transgenic models. It remains to be seen whether this mechanism is applicable to all ALS cases.

For cellular homeostasis, a fine balance between autophagosome formation and degradation needs to be maintained. Either over production of autophagosomes or decreased degradation of autophagosomes in neuronal cells can lead to impaired proteostasis and subsequent neurodegeneration [43]. To our knowledge, only one study has demonstrated autophagic regulation by ACM. Perucho et al. demonstrated that transfusion of ACM into an animal model of Huntington’s disease is sufficient to induce autophagy and regulate the expression of mutant huntingtin inclusions in vivo [35].

Our research demonstrates that cells treated with ALS patient ACM demonstrate significantly decreased levels of LC3-II. LC3-II is required for the selective autophagic degradation of p62 and targeted cargo. Decreased availability of LC3-II in response to increased autophagosome formation may result in accumulation of developing autophagosomes, disrupting the balance between autophagosome formation and degradation, leading to impaired proteostasis. Indeed, we observe increased numbers of p62 puncta awaiting degradation. p62 is regularly observed to be increased in ALS models [16], and we suggest that this phenomenon may be mediated by astrocytes.

Autophagic degradation of several ALS-related proteins, including SOD1 and TDP-43, is mediated via p62 and LC3B [37, 38]. Although no difference in TDP-43 expression was observed, a significant increase in SOD1 protein expression was demonstrated in response to ACM. Increased SOD1 cytoplasmic aggregations are often observed in patients, both with and without mutations within the *SOD1* gene [44]. Whether the increase in SOD1 protein expression in response to patient ACM is a precursor to the development of pathological insoluble aggregations will require further study. Additionally, it will be interesting to observe whether more prolonged treatment with patient ACM would alter the expression of other ALS-related proteins.

Activation of autophagy has been demonstrated to be beneficial to the survival of ALS animal models [21–24, 45]. This research aimed to determine whether activation of autophagy, in addition to ACM treatment, would modify the accumulation of p62 puncta. Rapamycin induces autophagy via inhibition of mTOR and activation of ULK1 [9], whereas the mechanism by which Trehalose modulates autophagy remains unclear [46]. We show that Rapamycin treatment decreases p62 accumulation in cells treated with patient ACM. No additive effect is observed in cells treated with control conditioned medium and Rapamycin. Conversely, treatment with Trehalose has no effect on p62 accumulation in cells treated with patient ACM. Surprisingly, in cells treated with both control ACM and Trehalose, we observe a stark increase in p62 accumulation. It is unclear why this occurs, but may be due to dual activation of autophagy by ACM and Trehalose. Regulation of autophagy in the presence of patient ACM may aid in the identification of pharmacological agents with therapeutic potential.

Although Rapamycin was able to ameliorate the increased accumulation of p62 in cells treated with patient ACM, no changes in SOD1 expression were observed following the same treatment. It remains to be determined whether this increase in SOD1 expression is mediated via impaired autophagy, and whether prolonged activation of autophagic mechanisms may counter the accumulation in cells treated with patient ACM.

Several reports have suggested that astrocytes mediate cell death by the secretion of toxic factors. Our results suggest that ACM may disrupt the balance between autophagosome formation and degradation, notably by decreased expression of LC3-II. The mechanism by which these cells fail to induce, or possibly inhibit, expression of this key autophagy protein is unclear, but may represent a significant factor contributing to impaired autophagy in ALS models. Future studies will focus on further elucidating the mechanisms by which patient ACM mediates impaired autophagy, and fully establishing cellular methodologies for investigating therapeutic agents using patient and control derived cells.

## Methods

### Generation of iPSCs

The iPS04c3, iPS21c1, iPS21cx and iPS31c8 lines were generated using STEMCCA lentivirus reprogramming kit (SCR544, Merck Millipore). . Briefly, 2.0 × 10^4^ fibroblasts on a γ-irradiated mouse embryonic feeder layer were transduced with the polycistronic lentivirus containing *OCT4*, *SOX2*, *KLF4* and *c-MYC* in the presence of 5 μg/ml Polybrene in complete fibroblast medium. Transduction was repeated after 24 h. On day 3 the medium was replaced with knockout replacement medium comprised of KnockOut™ DMEM, supplemented with 10% KnockOut™ Serum Replacement (Gibco), 0.1 mM MEM Non-Essential Amino Acids Solution (Gibco), 0.1 mM β-mercaptoethanol, 1 mM L-glutamine and 10 ng/ml basic fibroblast growth factor (Peprotech, UK). Media were changed daily. Colonies with an ES-like morphology were picked after day 21 of reprogramming and expanded for further characterization.

The iPSC1cx1, iPSC3c2 and iPS24c1 generated from fibroblasts using the oriP/EBNA1-based episomal vector from the Epi5™ Episomal iPSC Reprogramming Kit, containing the *OCT*, *SOX2*, *LIN28*, *L-MYC* and *KLF4* plus *mp53DD* with additional EBNA (Thermo Fisher, UK). Cells were transfected via nucleofection using program U-023. 5.0 × 10^4^ fibroblasts were transfected per line and transferred to Geltrex™ coated wells. Cells were cultured in knockout replacement medium as listed above, supplemented with 100 ng/ml basic fibroblast growth factor for the following 14 days. On day 15 the medium was replaced with Essential 8™ Medium (Gibco) with daily change. Colonies appeared by day 12 and the first clones were isolated for expansion on day 21 of reprogramming.

iPSCs were maintained in Pluristem culture medium (Pluristem therapeutics) on plates coated with Geltrex (Thermo Fisher).

### Cell differentiation

To differentiate cells towards motor neurons, the protocol by Du et al. was followed [30]. For the generation of astrocytes, motor neuron progenitor cells generated using the protocol by Du et al. were cultured in DMEM/Glutamax (Life Technologies) supplemented with 10% FBS (Fisher Scientific). These cells were passaged 1:3 using trypsin/EDTA when 80% confluency was reached.

### HEK 293T cell culture

HEK293T cells were maintained in DMEM/Glutamax supplemented with 10% FBS. These were routinely passaged using trypsin/EDTA at a ratio of 1:10 when ~90% confluency was reached.

### Conditioned medium

To generate astrocyte conditioned medium (ACM), astrocytes were cultured to confluency at which point cells were washed three times with PBS and the medium replenished. ACM was collected after 5 days of culture, filtered through 0.45um syringe filter, and stored at −80 °C until use. For experiments of motor neuron viability, motor neuron medium was used for the preparation of ACM. For experiments on HEK293T cells, DMEM supplemented with 10% FBS was used for the preparation of conditioned medium.

### Culture with ACM

To assess the effects of ACM on target cells, conditioned medium obtained from individual iPSC-astrocyte lines was cultured on cells for 5 days, then cells were fixed using 4% paraformaldehyde for immunocytochemistry analysis, or protein was harvested for western blot analysis. To assess the effects of drug treatments, cells were treated with 200nM Rapamycin (Sigma, #R8781) or 100nM Trehalose (Sigma, #T9531) for the final 48 h of co-culture.

### Motor neuron viability assessment

To generate motor neurons, 10^6^ motor neuron progenitor cells were cultured in suspension using Aggrewell 400 plates (Stem Cell Technologies) to form uniform sized motor neuron progenitor spheres. These were cultured in suspension for 6 days. After 6 days, 30 spheres were placed into each well of a 24-well plate coated with Geltrex. Neurons were allowed to grow from spheres for 4 days prior to culture in ACM. Cells were cultured with ACM for a total of 5 days, after which cells were fixed for immunocytochemistry analyses and quantification.

### Immunocytochemistry

For immunocytochemistry analysis, cells were washed with PBS and fixed in 4% paraformaldehyde (Santa Cruz, sc-253236) for 15 min at room temperature. Cells were then washed twice with 0.1% Tween 20 (Sigma, P9416) followed by permeablising in 1% Triton X-100 (Sigma, T8787) for 30 min. Cells were then blocked in 10% goat serum (Sigma) with 1% BSA (Sigma, A2153) and 1% Triton X-100. After, cells were washed three times with PBS and incubated overnight with primary antibodies as follows: SSEA4, SOX2, TRA-180 and OCT4 (CST, #9656S, each 1:200 dilution), MNX1 (DSHB, 81.5C10, 1:50 dilution), TUJ1 (Sigma, T2200, 1:200), GFAP (Dako, Z033401-2, 1:200 dilution), p62 (Abcam - ab56416, 1:100). Hoechst was used to stain nuclei (BD Pharmingen, #561908). Secondary antibodies (Thermo Fisher cat: A-11001 and A-11037) were used at a dilution of 1:2000.

### Western blot analysis

For western blot analysis Protein was harvested using RIPA buffer (Sigma, R0278) supplemented with protease inhibitors (Santa Cruz, sc-29131). Protein concentration was calculated using BCA kit (Thermo Fisher Cat #23225). 10ug of protein was loaded on 12% SDS polyacrylamide gels. The following primary antibodies were used: SOD1 (Sigma, HPA001401, 1:1000 dilution), TDP-43 (CST - #3448, 1:1000 dilution), ATG12 (CST, #4180, 1:1000 dilution), BECLIN-1 (CST, #3495, 1:1000 dilution), pULK1 (CST, #5869, 1:1000 dilution), LC3B (CST, #12741, 1:1000 dilution), mTOR (CST - #2972, 1:1000 dilution), ATG3 (CST - #3415, 1:1000 dilution), β-Actin (Sigma, A3854, 1:50000 dilution). Secondary antibodies were used at a dilution of 1:2000 (CST, #7074 and #7076). Blots were detected using chemiluminescence substrate (Millipore Cat# WBKLS0500).

### Imaging and analyses

The Operetta imaging hardware and Harmony software (Perkin Elmer) were used to image and quantify cells/puncta. Nuclei were identified by staining with Hoechst and MNX1 (for motor neurons). After identifying cytoplasmic regions based on p62 expression, p62 puncta were identified using the spot finder tool. For the quantification of motor neurons the entirety of each well was visualized and MNX1 positive cells were quantified. For the quantification of p62 puncta a minimum of 30 fields of view were used for each condition.

### Statistical analyses

All statistical analyses were performed using Graph Pad Prism. *p < 0.05 and **p < 0.01 were deemed statistically significant.

## Abbreviations

ACM: Astrocyte conditioned medium
ALS: Amyotrophic Lateral Sclerosis
ATG: Autophagy related proteins
iPSCs: Induced pluripotent stem cells
